# Transfer Entropy

**DOI:** 10.3390/e20040288

**Published:** 2018-04-16

**Authors:** Deniz Gençağa

**Affiliations:** Department of Electrical and Electronics Engineering, Antalya Bilim University, Antalya 07190, Turkey; deniz.gencaga@antalya.edu.tr

**Keywords:** transfer entropy, causal relationships, entropy estimation, statistical dependency, nonlinear interactions, interacting subsystems, information-theory, Granger causality, mutual information, machine learning, data mining

Statistical relationships among the variables of a complex system reveal a lot about its physical behavior. Therefore, identification of the relevant variables and characterization of their interactions are crucial for a better understanding of a complex system. Correlation-based techniques have been widely utilized to elucidate the linear statistical dependencies in many science and engineering applications. However, for the analysis of nonlinear dependencies, information-theoretic quantities, such as Mutual Information (MI) and the Transfer Entropy (TE), have been proven to be superior. MI quantifies the amount of information obtained about one random variable, through the other random variable, and it is symmetric. As an asymmetrical measure, TE quantifies the amount of directed (time-asymmetric) transfer of information between random processes and therefore is related to the measures of causality.

In the literature, the Granger causality has been addressed in many fields, such as biomedicine, atmospheric sciences, fluid dynamics, finance, and neuroscience. Despite its success in the identification of couplings between the interacting variables, the use of structural models restricts its performance. Unlike Granger causality, TE is a quantity that is directly estimated from data and it does not suffer from such constraints. In the specific case of Gaussian distributed random variables, equivalence between TE and Granger causality has been proven. 

The estimation of TE from data is a numerically challenging problem. Generally, this estimation depends on accurate representations of the probability distributions of the relevant variables. Histogram and kernel estimates are two common ways of estimating probability distributions from data. TE can be expressed in terms of other information-theoretic quantities, such as Shannon entropy and MI, which are functions of the probability distributions of the variables. Therefore, it is prone to errors due to the approximations of probability distributions. Moreover, many TE estimation techniques suffer from the bias effects arising from the algebraic sums of other information-theoretic quantities. Thus, bias correction has been an active research area for better estimation performance. Methods such as Symbolic TE and the Kraskov-Stögbauer-Grassberger (KSG) algorithm are among the other techniques used to estimate TE from data. The efficient estimation of TE is still an active research area.

Most of these techniques have been proposed to solve specific problems in diverse applications. Hence, a method proposed for the solution of one application might not be the best for another. This Special Issue has been organized to collect distinctive approaches in one publication, as a reference tool for the theory and applications of TE. 

The contributions are categorized into two sections: the methods and the applications. 

## 1. Methods and Theory

The first section begins with the presentation of a recipe to estimate the information flow in dynamical systems [1]. In their work, Gencaga et al. propose a Bayesian approach to estimate TE and apply a set of methods together as an accuracy cross-check to provide a reliable mathematical tool for any given dataset. The work of Zhu et al. [2] proposes a k-Nearest Neighbor approach to estimate TE and demonstrates its effectiveness as an extension of the KSG MI estimator.

The methodological section continues with the analytical derivations of the TE expressions for a class of non-Gaussian distributions. Here, Jafari-Mamaghani and Tyrcha [3] provide the expressions of TE in the cases of multivariate exponential, logistic, Pareto (Type I-IV), and Burr distributions. Next, Nichols et al. elaborate on the linearized TE for continuous and coupled second-order systems and they derive an analytical expression for time-delayed transfer entropy (TDTE) [4]. They conclude with an alternative interpretation of TE, which can be viewed as a measure of the ability of a given system component to predict the dynamics of another. Coupling between random processes is also explored by Hahs and Pethel [5], where the TE is computed over multiple time lags for multivariate Gaussian autoregressive processes. In two examples, they demonstrate the change in TE as a response of variations in the correlation and coupling coefficient parameters. The case of coupling dynamics with time-varying dependencies is investigated by Gómez-Herrero et al. [6] if access to an ensemble of independent repetitions of time series is available. They estimate combinations of entropies and detect time-varying information flow between dynamical systems using the ensemble members. 

The relation between Granger causality and directed information theory is discussed next in the review paper of Amblard and Michel [7], in which they focus on conditional independence and causal influences between stochastic processes. In addition to the link between directed information and hypothesis testing, instantaneous dependencies are emphasized to be different than dependencies on past values.

The next two papers demonstrate two new interpretations of TE. First, motivated by the relativistic effects on the observation of information dynamics, Lizier and Mahoney bring a new explanation of a local framework for information dynamics [8]. Second, Prokopenko et al. present a thermodynamic interpretation of TE near equilibrium and emphasize the nuance between TE and causality [9]. The methodological section ends with the comparisons of Papana et al. where they study direct causality measures in multivariate time series by simulations. The authors compare measures such as the conditional Granger causality index, partial Granger causality index, partial directed coherence, partial TE, partial symbolic TE, and partial MI on mixed embedding. Simulations include stochastic and chaotic dynamical systems with different embedding dimensions and time series lengths [10].

## 2. Applications

In this section, we present six contributions on the applications of TE. In the first paper, Faes et al. introduce a tool for reliably estimating information transfer in physiological time series using compensated TE [11]. This tool provides a set of solutions to the problems arising from the high dimensionality and small sample size, which are frequently encountered in entropy estimations of cardiovascular and neurological time series. Next, Materassi et al. elucidate a different normalized TE and use it to detect the verse of energy flux transfer in a synthetic model of fluid turbulence, namely the Gledzer-Ohkitana-Yamada shell model [12]. They emphasize the superior performance compared to those of the traditional methods. Applications continue with a paper on network inference by Ai [13]. The author addresses a TE-based framework to quantify the relationships among topological measures and provides a general approach to infer a drive-response structure in a complex network. This work is followed by two financial applications. The first contribution is authored by Li et al., in which a TE-based method is developed to determine the interbank exposure matrix between banks and the stability of the Chinese banking system is evaluated by simulating the risk contagion process [14]. In the second application, Sandoval Jr. uses the stocks of the 197 largest companies in the world and explores their relationships using TE [15]. This Special Issue ends with the presentation of the theory and applications of the Liang-Kleeman information flow [16]. Here, Liang points out the importance of information flow as a potential measure of the cause and effect relation between dynamical events and presents applications on the Baker transformation, Henon map, truncated Burgers-Hopf system, and Langevin equation.

This Special Issue demonstrates the importance of information-theoretic quantities in the analysis of the statistical dependencies between the variables of a complex system. Unlike correlation and MI, TE is shown to be effective for the detection of directional interactions, which are closely related to cause and effect relationships. The examples demonstrate the difficulties in estimating information-theoretic quantities from data and present approaches to overcome these problems.

In this Special Issue, we have collected 16 outstanding papers by the experts in the field. I would like to express our special thanks to each researcher and anonymous referee for their invaluable contributions. I would also like to thank the Editor-in-Chief, Prof. Kevin H. Knuth, for his encouragement during the organization of this Special Issue. My grateful thanks are also extended to the members of the editorial board and the editorial assistants of the Entropy Journal for their support. Last, but not least, I would like to thank MDPI Books for giving me the opportunity to publish this Special Issue.

We are excited to present this Special Issue as a reference for the theory and applications of transfer entropy and we hope that this publication contributes to novelties in all disciplines of research and development.

## References

[1] Gencaga D., Knuth K., Rossow W. (2015). A Recipe for the Estimation of Information Flow in a Dynamical System. Entropy.

[2] Zhu J., Bellanger J., Shu H., Le Bouquin Jeannès R. (2015). Contribution to Transfer Entropy Estimation via the k-Nearest-Neighbors Approach. Entropy.

[3] Jafari-Mamaghani M., Tyrcha J. (2014). Transfer Entropy Expressions for a Class of Non-Gaussian Distributions. Entropy.

[4] Nichols J., Bucholtz F., Michalowicz J. (2013). Linearized Transfer Entropy for Continuous Second Order Systems. Entropy.

[5] Hahs D., Pethel S. (2013). Transfer Entropy for Coupled Autoregressive Processes. Entropy.

[6] Gómez-Herrero G., Wu W., Rutanen K., Soriano M., Pipa G., Vicente R. (2015). Assessing Coupling Dynamics from an Ensemble of Time Series. Entropy.

[7] Amblard P., Michel O. (2013). The Relation between Granger Causality and Directed Information Theory: A Review. Entropy.

[8] Lizier J., Mahoney J. (2013). Moving Frames of Reference, Relativity and Invariance in Transfer Entropy and Information Dynamics. Entropy.

[9] Prokopenko M., Lizier J., Price D. (2013). On Thermodynamic Interpretation of Transfer Entropy. Entropy.

[10] Papana A., Kyrtsou C., Kugiumtzis D., Diks C. (2013). Simulation Study of Direct Causality Measures in Multivariate Time Series. Entropy.

[11] Faes L., Nollo G., Porta A. (2013). Compensated Transfer Entropy as a Tool for Reliably Estimating Information Transfer in Physiological Time Series. Entropy.

[12] Materassi M., Consolini G., Smith N., De Marco R. (2014). Information Theory Analysis of Cascading Process in a Synthetic Model of Fluid Turbulence. Entropy.

[13] Ai X. (2014). Inferring a Drive-Response Network from Time Series of Topological Measures in Complex Networks with Transfer Entropy. Entropy.

[14] Li J., Liang C., Zhu X., Sun X., Wu D. (2013). Risk Contagion in Chinese Banking Industry: A Transfer Entropy-Based Analysis. Entropy.

[15] Sandoval L. (2014). Structure of a Global Network of Financial Companies Based on Transfer Entropy. Entropy.

[16] Liang X. (2013). The Liang-Kleeman Information Flow: Theory and Applications. Entropy.

